# Thickness-Dependent NIR LSPR of Curved Ag/TiS_2_ Bilayer Film

**DOI:** 10.3390/molecules25194551

**Published:** 2020-10-05

**Authors:** Yongjun Zhang, Fan Zhang, Yaxin Wang

**Affiliations:** 1 School of Material and Environmental Engineering, Hangzhou Dianzi University, Hangzhou 310012, China; yjzhang@hdu.edu.cn; 2 Department of Physics, Southeast University, Nanjing 211189, China; 3 Key Laboratory of Functional Materials Physics and Chemistry, Ministry of Education, College of Physics, Jilin Normal University, Changchun 130103, China

**Keywords:** localized surface plasmon resonances (LSPR), NIR region, carrier density

## Abstract

We demonstrated that the localized surface plasmon resonance (LSPR) features of Ag/TiS_2_ nanostructures were dependent on the sublayer thickness. The Ag/TiS_2_ bilayer film was obtained by the self-assembly method and magnetron sputtering. The thickness was controlled by changing the sputtering time when the sputtering powers were the same. When the Ag thickness decreased from 50 nm to 5 nm, the LSPR was tuned from the visible region to the Near Infrared (NIR) region. When the TiS_2_ thickness decreased from 60 nm to 2 nm, the LSPR shifted from the IR to NIR region. Analysis showed the thickness changes of Ag and TiS_2_ resulted in the changed carrier density, which led to the thickness-dependent shift of the LSPR.

## 1. Introduction

For nanostructured noble metals, the collective resonances of the free carriers of the sufficient density leads to localized surface plasmon resonances (LSPR) [1,2,3]. LSPR had been reported in noble metals for decades, which is widely used in catalysis, information storages, biosensors and surface-enhanced Raman scattering [4,5,6,7,8]. In recent studies, the LSPR of noble metal nanoparticles was tuned by the size, shape, composition, dielectric environment, and so on [9,10,11,12]. According to the Drude model, [13] when the carrier density of noble metals is around 10^22^, LSPR was calculated to be in the visible region. In addition to noble metals, LSPR was also found in some doped semiconductors, in which the high-level doping increased carrier density and controlled the position of the LSPR bands. Hsu et al. [14] reported that LSPR for Cu_2-x_S controlled by the geometric aspect ratio increased the free carrier density. In Jacob’s work [15], ZnO nanocrystals showed an infrared (IR) LSPR via the photoexcitation of electrons. Since then, LSPR is no longer regarded as a unique property of noble metals, but optical signatures of carrier collection. In recent years, metal composites have been synthesized, which produced a lot of new characteristics. In Ag–FeS [16] and Ag/Cu_2_S [17] nanocomposites, which were based on the interaction between the noble and semiconductors, the electronic environment around the nanostructure changed with different thickness, and the coupling between two particles was dominant, which led to the displacement of LSPR. More and more nanostructures with LSPR properties are widely used in cancer detection, photodetection, photodegradation, and so on [18,19,20,21].

Zhu et al. [22] first observed the existence of near-IR (NIR) LSPR characteristics in semi-metallic TiS_2_, which proved the possible wide applications of NIR LSPR of semi-metallic TiS_2_. TiS_2_ is one of the transition metal dichalcogenides (TMD), which has good applications in energy materials [23,24,25,26,27] and biomolecule detection [28,29]. The energy band structure of TiS_2_ is similar to that of metals, and TiS_2_ has a high carrier concentration and mobility. In addition, the carrier density of bulk TiS_2_ is about 10^21^ [22]. Unlike noble metals, the carrier density of TiS_2_ could be realized by controlling the thickness of semi-metallic TiS_2_, which can appreciably tune LSPR dynamically. In our previous works [30], we prepared the curved TiS_2_–Ag nanostructure on a PS array, which could regulate the position of LSPR from the visible region to the NIR region as the carrier density decreased. Therefore, it was possible to widen the LSPR wavelength range of TiS_2_ with changed carrier density on polystyrene bead arrays. LSPR is sensitive to the thickness and morphology, so the curved Ag/TiS_2_ bilayer films with the different structures and compositions have various properties.

Here, we prepared curved Ag/TiS_2_ nanostructures on a two-dimensional polystyrene (PS) bead array by magnetron sputtering technology, in which LSPR could be regulated from the visible region or IR region to the NIR region by changing the Ag/TiS_2_ thickness. The PS arrays worked as curved substrates, inducing various Ag/TiS_2_ thicknesses, which made the LSPR bands of the Ag/TiS_2_ nanostructures localized in the NIR wavebands. This tunable LSPR characteristic provides curved Ag/TiS_2_ nanostructures with more applications in different fields, for example, biological and communication windows.

## 2. Experimental Section

### 2.1. Materials

The mono-disperse polystyrene (PS) colloid with size 200 nm and the density 1.05 g/cm^3^ was purchased from the Duke Scientific Corporation. NH_4_OH (25%) and H_2_O_2_ (30%) were purchased from Sigma-Aldrich Co. Ltd. (Beijing, China) and the Sino-Pharm Chemical Reagent Co. Ltd. (Shanghai, China). 4-Aminothiophenol (4-ATP) was purchased from Sigma-Aldrich Co. Ltd. The purity of Ag and TiS_2_ was 99.99%, and were purchased from Beijing Jing Mai Mstar Technology Co. Ltd., Beijing, China. The resistivity of the deionized water was 18.0 MΩ cm and the silicon wafers with the (100) crystal orientation were purchased from the Hefei Kejing Materials Technology Co. Ltd., Hefei, China.

### 2.2. Preparation of Polystyrene (PS) Arrays

The single-layer PS sphere arrays were prepared by the self-assembly method. The suitable size of silicon wafers was boiled in a mixed solution of H_2_O, NH_4_OH. and H_2_O_2_ (6:1:2) for 5–10 min. Then, the silicon wafers were ultrasonically washed three times with deionized water and absolute ethanol. Finally, the silicon wafers were stored in deionized water. The volume ratio 1:1 of PS solution to ethanol was dropped on a large silicon wafer using a pipetting tip. The large silicon wafers were slanted into water. Driven by the water, the PS spheres on the silicon wafer formed single layer colloidal sphere arrays on the water surface. Clean silicon wafers were used to pick up the single layer film on the water surface. The silicon wafers with PS arrays were dried under natural conditions to form a 2D ordered array on the silicon substrates.

### 2.3. Preparation of SERS Active Substrates

**Figure 1:**
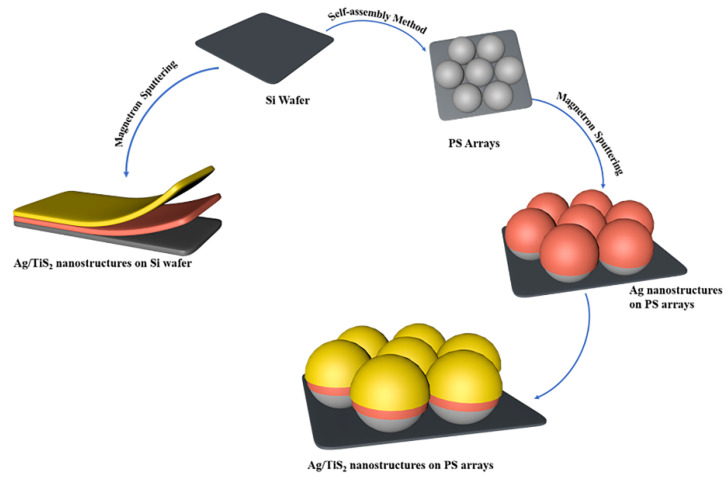
The preparation schematic diagram of the Ag/TiS_2_ nanostructure on Si wafers and PS arrays.

Figure 1 shows the schematic diagram of the Ag/TiS_2_ nanostructure on PS arrays and Si wafers. The preparation process of Ag/TiS_2_ films was carried out in a magnetron deposition system. First, the two-dimensional ordered arrays with a diameter of 200 nm were fabricated by the self-assembly technique. Then, the Ag films were sputtered on the PS arrays. Finally, the TiS_2_ film was sputtered on the Ag film that formed Ag/TiS_2_ curved nanostructures. The preparation method and conditions of the Ag/TiS_2_ flat planar nanostructures were the same as the curved nanostructure. Thus, the single Ag (5, 10, 20, 30, 50 nm) nanostructures and the single TiS_2_ (2, 5, 10, 20, 30, 60 nm) nanostructures on PS arrays were prepared as were Ag 10 nm/TiS_2_ (2, 5, 10, 20, 30, 60 nm) nanostructures and the Ag 5 nm/TiS_2_ (2, 5, 10, 20, 30, 60 nm) nanostructures of curved and flat planar films.

### 2.4. Characterization of Substrates

The Ag/TiS_2_ bilayer film was performed by the magnetron system (JGP-560C, Shenkeyi, Shenyang, China) with vacuum 2 × 10^−4^ Pa. The working pressure was 0.6 Pa. The TiS_2_ film thickness was 2, 5, 10, 20, 30, and 60 nm, and the growth rate was 2 nm/min. The Ag film sputter power was 21.6 W and the growth rate was 10 nm/min. The performance characterization methods used in the experiment mainly included scanning electron microscope (SEM, JEOL 7800F, Tokyo, Japan) with accelerating voltage of 200 kv. Raman spectra were measured on a Renishaw Raman confocal microscopy spectrometer (model 2000, Renishaw, London, UK), and the excitation wavelength was 633 nm. Transmission electron microscope (TEM, JEM-2100HR, JEOL LTD., Tokyo, Japan) and UV–Vis–NIR spectra with a Shimadzu UV-3600 plus spectrophotometer (Shimadzu, Kyoto, Japan) and Hall Effect were also carried out.

## 3. Results and Discussion

**Figure 2:**
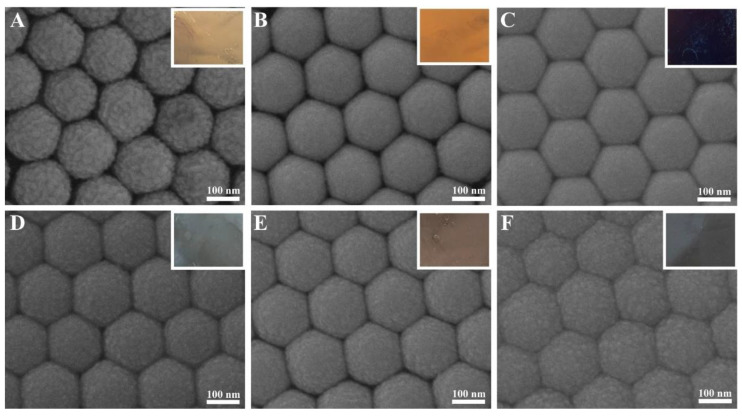
Scanning electron microscope (SEM) images of the Ag 10 nm/TiS_2_ (t nm) nanostructures on PS. (**A**) 2, (**B**) 5, (**C**) 10, (**D**) 20, (**E**) 30, and (**F**) 60 nm. The insets are the corresponding optical photos.

**Figure 3:**
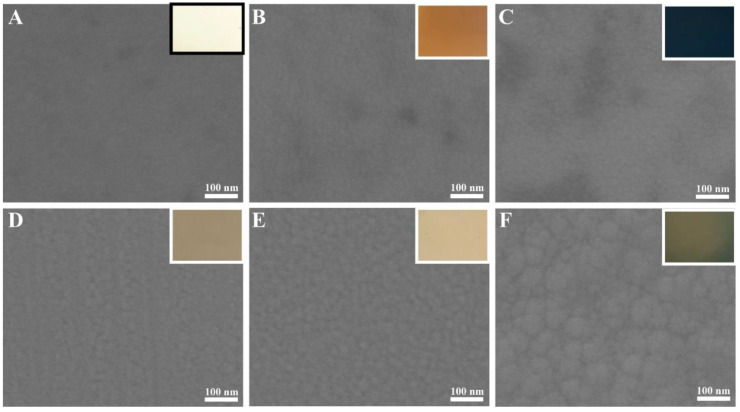
SEM images of Ag 10 nm/TiS_2_ (t nm) nanostructures on Si wafers. (**A**) 2, (**B**) 5, (**C**) 10, (**D**) 20, (**E**) 30, and (**F**) 60 nm. The insets are the optical photos.

The morphological characteristics of Ag 10 nm/TiS_2_ (t = 2, 5, 10, 20, 30, 60 nm) nanostructures are shown in Figure 2 and Figure 3. After sputtering 2 nm TiS_2_ on 10 nm Ag film surfaces, the curved films tended to form round particles because the TiS_2_ layer did not show good compatibility with Ag in Figure 2A. When the thickness of TiS_2_ was 5 nm, the nanocap surfaces of the Ag 10 nm/TiS_2_ 5 nm nanostructures became smooth. While the TiS_2_ thickness increased, the effects of incompatibility between Ag and TiS_2_ film morphology were reduced. When the TiS_2_ thickness increased from 20 nm to 60 nm, the surface roughness of the nanocaps gradually increased due to the growth of the TiS_2_ nanoparticles. The nanostructures’ lateral size became larger, and the gaps became smaller. The adjacent nanocaps were closely arranged and extruded to form hexagonal structures. The corresponding optical photos in the insets showed different reflected colors, which were ascribed to the different thickness and surface morphologies of Ag/TiS_2_ film. Furthermore, the uniform diffraction color indicated that the high-ordered PS colloid sphere arrays and uniform film had formed. Figure 3 shows the SEM of the flat film of the Ag 10 nm/TiS_2_ (t = 2, 5, 10, 20, 30, 60 nm) nanostructures. As the TiS_2_ thickness increased, the surface roughness of the nanocaps showed similar changes to the Ag 10 nm/TiS_2_ nanostructures on the PS arrays. The uniform color of the insets showed that the films on the plane were uniform.

**Figure 4:**
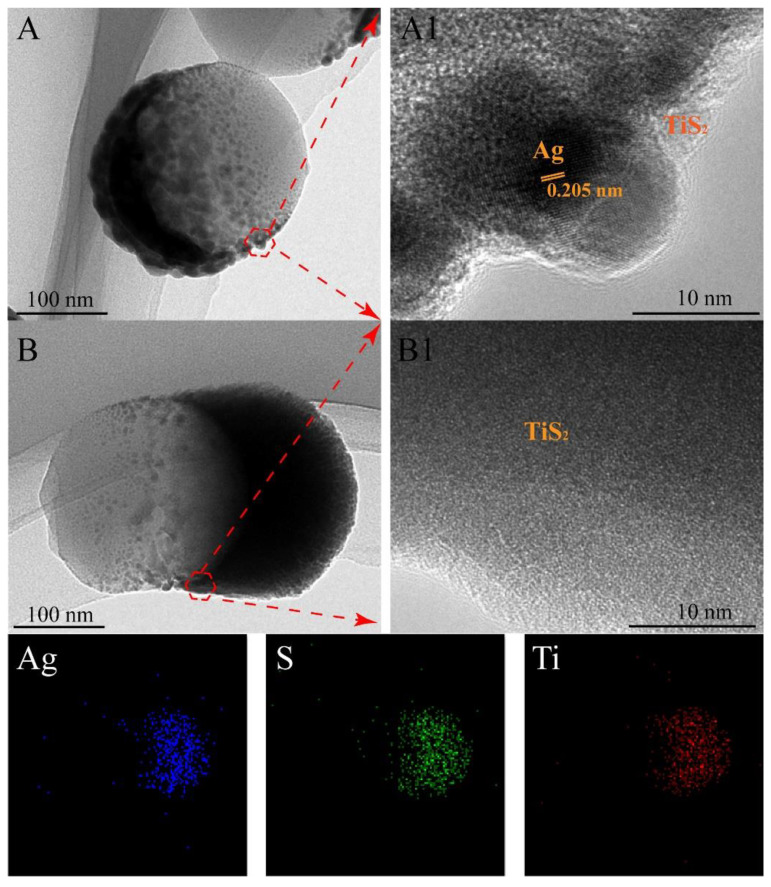
TEM and HRTEM images of (**A**,**A1**) Ag 10 nm/TiS_2_ (2 nm) nanostructures and (**B**,**B1**) Ag 10 nm/TiS_2_ (30 nm) nanostructures. The EDS elemental mapping is shown in (**B**).

Figure 4 shows the TEM, HRTEM, and EDS mapping images of the Ag 10 nm/TiS_2_ (t = 2 nm, 30 nm) nanostructures. In Figure 4A,B, the TEM images showed that Ag/TiS_2_ composite films covered the PS spheres like the “nanocap”. The sizes of the thickest part were about 12 nm and 40 nm, corresponding to the sums of the Ag thickness and TiS_2_ thickness, respectively. In comparison with Ag 10 nm/TiS_2_ 2 nm (Figure 4A), Ag 10 nm/TiS_2_ 30 nm (Figure 4B) showed obvious anisotropic growth due to the shadow effect of the neighboring PS beads. The HRTEM images of Ag 10 nm/TiS_2_ (2 nm, 30 nm) correspond to the red marked area in Figure 4A,B. In Figure 4A1, the transparent materials at the edge of Ag were amorphous TiS_2_, and the lattice space of Ag was 0.205 nm, in agreement with the (100) plane. Ag was not observed in the HRTEM images (Figure 4B1), which could be attributed to the complete coverage by TiS_2_ with a thickness three times that of the Ag thickness. EDS elemental analysis showed the Ag, S, and Ti elements, which were mainly distributed in the nanocaps. Ag elements were mainly distributed between the bottom of the nanocaps and the top of the PS. S and Ti elements were distributed across all nanocaps, which confirmed the Ag film under the TiS_2_ film and the uniform distribution of TiS_2_ on the Ag nanocaps.

**Figure 5:**
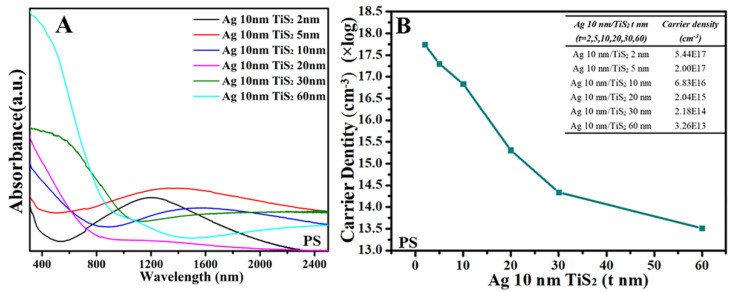
(**A**) UV–Vis–NIR spectra and (**B**) Hall effect for the Ag 10 nm/TiS_2_ (t = 2, 5, 10, 20, 30, 60 nm) nanostructures on PS, and the table inset is the original data.

Figure 5 shows the UV–Vis–NIR spectra for Ag 10 nm/TiS_2_ (t nm) nanostructures on PS. All the Ag 10 nm/TiS_2_ (t nm) nanostructures had absorbance from the UV to NIR region, which indicated that the carrier was collective oscillation in the substrates under the irradiation of UV–Vis–NIR light. When the TiS_2_ thickness increased, the carrier density always decreased, which led to the red-shift of absorbance bands from the visible to NIR region, even the IR region, as shown in Figure 5. Figure S1 is the UV–Vis–NIR spectra of single TiS_2_. The LSPR peaks of TiS_2_ red-shifted and widened as the TiS_2_ thickness increased, and the carrier density also decreased. The LSPR peaks of TiS_2_ red-shifted and widened as the TiS_2_ thickness increased, and the carrier density also decreased from 10^16^ to 10^13^ in Figure S1B. The sensitivity of Hall effect detection for free electrons and high carrier concentration was low, so the carrier concentrations were far less than the normal value of 10^20^~10^21^ cm^−3^. On the other hand, the very thin TiS_2_ films sputtered on the PS arrays led to the destroyed continuity and electrical conductivity of TiS_2_. According to previous reports, the decrease in carrier concentration would cause the red-shift of the LSPR, which resulted in the red-shifted maximum LSPR wavelength ($\lambda_{max}$) with the carrier concentration decreased [22]. While the carrier density of Ag 10 nm/TiS_2_ (t nm) nanostructures decreased, the $\lambda_{max}$ was red shifted. For Ag 10 nm/TiS_2_ (t nm) nanostructures, the optical absorbance bands were mainly composed of absorbance bands of TiS_2_ and synergistic effects of TiS_2_ and Ag so that the Ag 10 nm/TiS_2_ (t nm) nanostructures showed the widened absorbance bands (Figure 5A).

**Figure 6:**
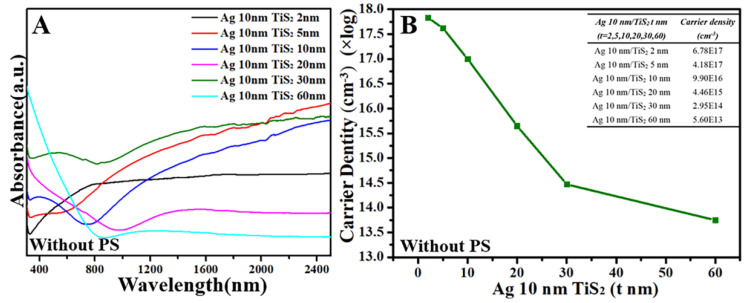
(**A**) UV–Vis–NIR spectra and (**B**) Hall effect for the Ag 10 nm/TiS_2_ (t nm) nanostructures without PS, and the table inset is the raw data.

Figure 6 is the UV–Vis–NIR spectra and Hall effect measurement of flat planar Ag 10 nm/TiS_2_ (t nm) nanostructures. When the thickness of TiS_2_ increased, the absorption bands showed a red-shift from the NIR to IR region as the carrier density decreased. The maximum absorption bands in the IR region could not be observed, which were beyond the detectable wavelength range of our instrumentation. However, the carrier density of the Ag 10 nm/TiS_2_ nanostructures without PS arrays (Figure 6) was slightly more than that on the PS arrays (Figure 5) because of the good continuity and electrical conductivity of plane films.

**Figure 7:**
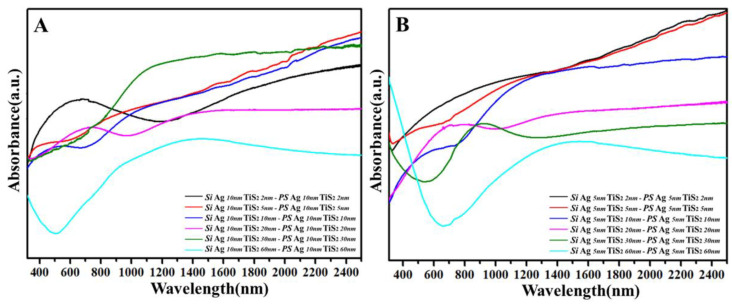
The different spectra of UV–Vis–NIR spectra for (**A**) Ag 10 nm/TiS_2_ (t nm) nanostructures and (**B**) Ag 5 nm/TiS_2_ (t nm) nanostructures.

In order to prove the effect of curved films, the absorption bands of flat Ag 10 nm/TiS_2_ (t nm) nanostructures and curved Ag 10 nm/TiS_2_ (t nm) nanostructures were compared. For curved Ag 10 nm/TiS_2_ (t nm) nanostructures, the absorption bands were situated around 1200 nm to 2400 nm. However, the absorption bands of flat Ag 10 nm/TiS_2_ (t nm) nanostructures were beyond the detectable wavelength range of instrumentation, so the optical absorption was localized at the IR region. Figure 7A shows the different UV–Vis–NIR spectra for Ag 10 nm/TiS_2_ nanostructures with and without PS arrays. With the increased TiS_2_ thickness, the absorbance peaks first red-shifted from the visible region to the NIR region, and then still in the NIR region. When the TiS_2_ thickness was 2, 5, and 10 nm, the curvature of every nanocap decreased, which led to the absorbance peaks red-shifting from the visible region to the NIR region. While the thickness was 20, 30, and 60 nm, the surface of the PS arrays gradually flattened, the absorbance peaks did not shift, and were located in the NIR region. When the Ag 10 nm/TiS_2_ nanostructures on the PS arrays were excited by light, the absorption bands of the PS arrays in the NIR region could couple with the Ag 10 nm/TiS_2_ nanostructures, which made the LSPR of the patterned structures in the NIR region, even if the carrier concentration of the Ag 10 nm/TiS_2_ nanostructures was as low as 10^14^. The curved shapes are important for LSPR in the NIR region. Due to the influence of the size and shape, the LSPR of the curved structures was adjustable. This was also an important reason as to why the array structures were superior to the flat structures. In order to validate the relationship between thickness and LSPR, the LSPR and carrier density of the Ag 5 nm/TiS_2_ (t nm) nanostructures are shown in Figures S2 and S3. The changed rule of LSPR and carrier density in these systems was also found, even if Ag 5 nm was chosen for the Ag/TiS_2_ nanostructures (Figure 7B, Figures S2 and S3). Therefore, the changes in the UV–Vis–NIR spectra primarily derived from the intrinsic factors of the Ag/TiS_2_ (t nm) systems such as carrier density, dielectric environment, and shape.

**Figure 8:**
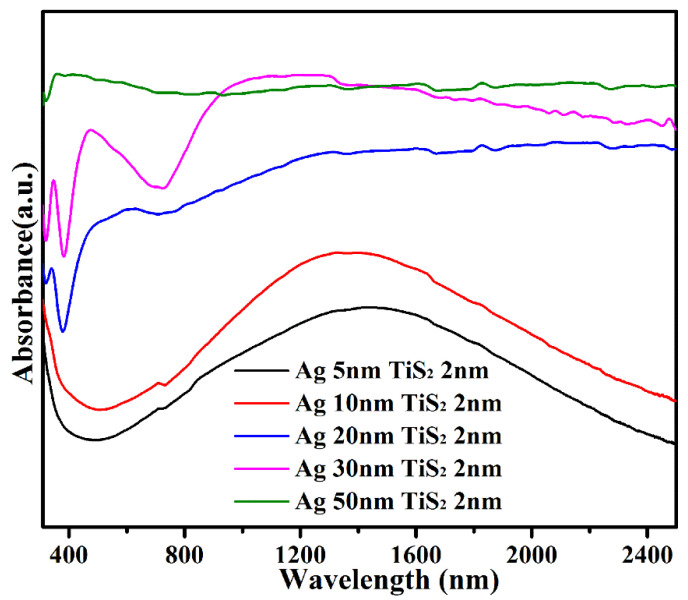
UV–Vis–NIR spectra for the Ag (t = 5, 10, 20, 30, 50 nm)/TiS_2_ (2 nm) nanostructures.

The UV–Vis–NIR spectra for the single Ag films are shown in Figure S4. The peak at 320 nm was the intrinsic peak of Ag, and peaks around 400–2400 nm corresponded to the plasmon resonance [31]. As Ag thickness increased, the plasmon resonance peaks of Ag around 400–2400 nm showed a blue-shift and became narrow due to the dielectric constant effect of surrounding medium. Compared with the single Ag (Figure S4), the LSPR bands of Ag (t nm)/TiS_2_ 2 nm nanostructures were significantly red shifted and relatively broad as the thickness of Ag decreased (Figure 8). For Ag and TiS_2_ composite films, the plasma resonance oscillations were coupled. Beyond that, the electromagnetic coupling enhanced the polarizability of the electron cloud and reduced the resonance energy of plasmon, which led to the red-shift and widened LSPR.

**Figure 9:**
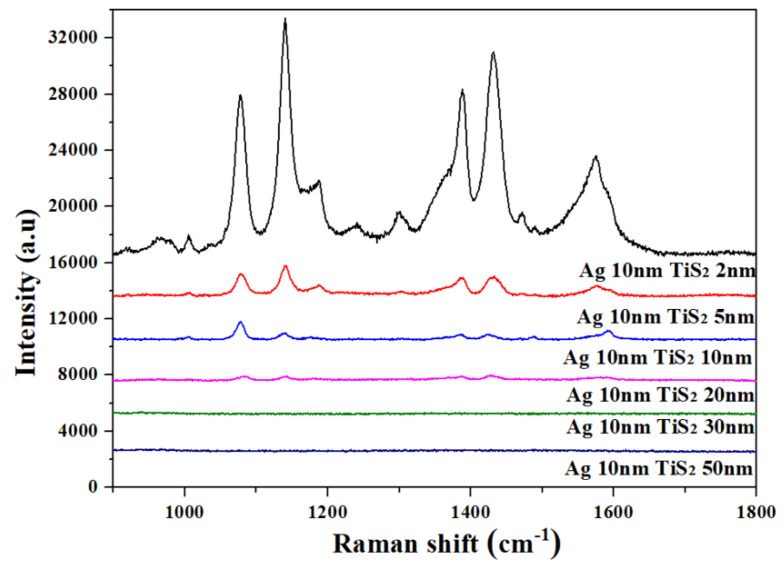
SERS spectra of Ag 10 nm/TiS_2_ (t = 2, 5, 10, 20, 30, 60 nm) nanostructures on PS.

The Raman spectra of the curved Ag 10 nm/TiS_2_ (t nm) nanostructures were measured in Figure 9. The excitation wavelength was 633 nm and the concentration of the 4-ATP probe molecule was 10^−3^ mol/L. In Figure 9, the Ag 10 nm/TiS_2_ (2, 5, 10 nm) nanostructures all showed characteristic peaks of PATP, in which the curved film could enhance the signals of PATP. The SERS signals of the Ag 10 nm/TiS_2_ (t nm) nanostructures were the strongest when the thickness of TiS_2_ was 2 nm. This was due to the SERS that majored from the LSPR of Ag and the “hot spots” of the adjacent nanocaps [32,33]. The Ag layer was below the TiS_2_ layer, and the TiS_2_ layer had a shielding effect on the LSPR of Ag, so the strength of Ag LSPR decreased exponentially with the thickness of the TiS_2_ layer [34]. As the TiS_2_ thickness increased, the contribution of Ag LSPR to the enhancement of the electromagnetic field decreased, and the SERS signal intensity gradually decreased.

## 4. Conclusions

The Ag/TiS_2_ nanostructures could achieve bidirectional regulation from visible region or IR region to NIR region. When the thickness of Ag decreased, the LSPR shifted from visible region to the NIR region for the curved Ag (t nm)/TiS_2_ 2 nm nanostructures. With the decreased thickness of TiS_2_, the LSPR of the curved Ag/TiS_2_ (t nm) nanostructures moved from the IR to the NIR region. For the Ag/TiS_2_ film on the PS arrays, the curved structures were adjusted for LSPR in the NIR region, even if carrier density was as low as 10^14^ for the curved Ag/TiS_2_ nanostructures. The Raman signals came from the LSPR of Ag in the “hot spots” of the adjacent nanocaps.

## Supplementary Materials

The following are available online.
