# Supplementary material for: Thickness-Dependent NIR LSPR of Curved Ag/TiS2 Bilayer Film
Source: Molecules. 2020 Oct 5;25(19):4551. doi: 10.3390/molecules25194551 (PMC7583025; doi:10.3390/molecules25194551)
Supplement: Supplementary file 1 [file molecules-25-04551-s001.pdf]

Thickness-Dependent NIR LSPR of Curved Ag/TiS_2 Bilayer Film

Yongjun Zhang [a], Fan Zhang [b,c*] and Yaxin Wang [a,*]

[a] School of Material and Environmental Engineering, Hangzhou Dianzi University, Hangzhou 310012, China.

[b] Department of Physics, Southeast University, Nanjing 211189, China

[c] Key Laboratory of Functional Materials Physics and Chemistry, Ministry of Education, College of Physics, Jilin Normal University, Changchun 130103, China

Correspondence: [*]E-mail: 230198829@seu.edu.cn; [*]E-mail: yaxinwang@hdu.edu.cn

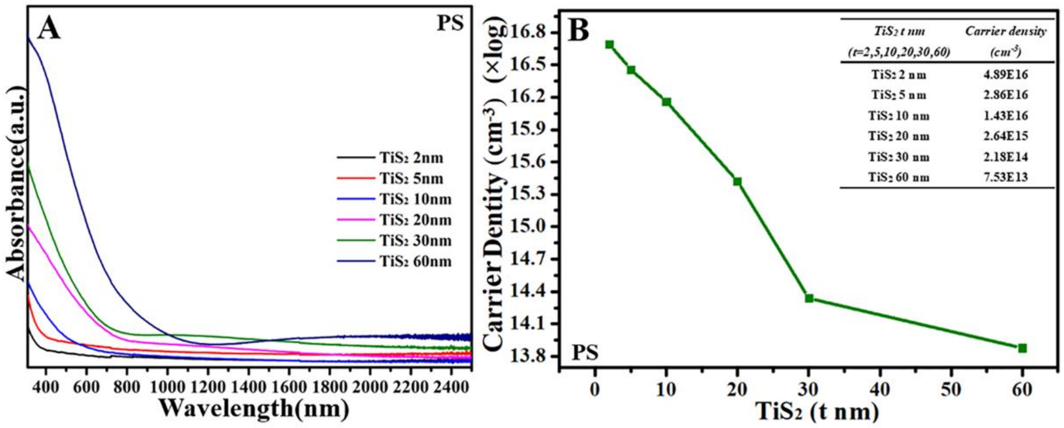

Figure S1. (A)UV-vis-NIR spectra, (B) Hall effect for TiS_2 (t nm) nanostructures on PS.

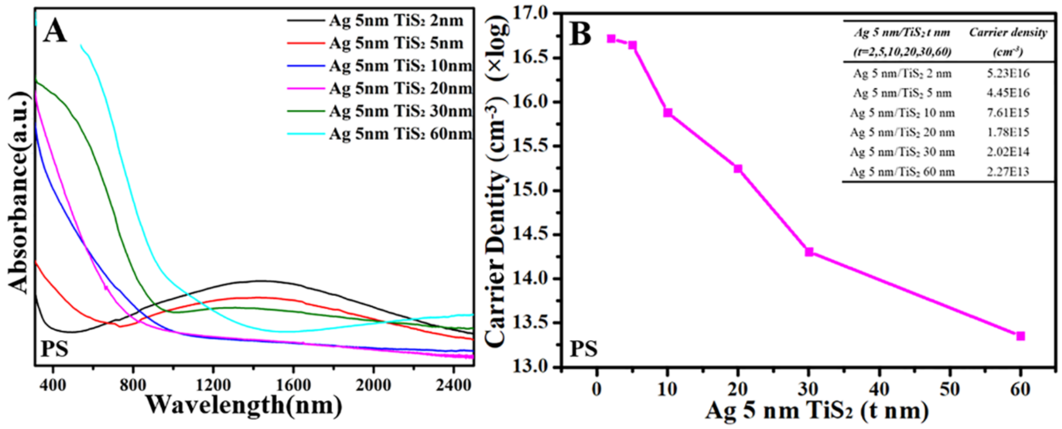

Figure S2. (A) UV-vis-NIR spectra and (B) Hall effect for Ag 5 nm/TiS_2 (t nm) nanostructures on PS, and the table of inset is the raw data.

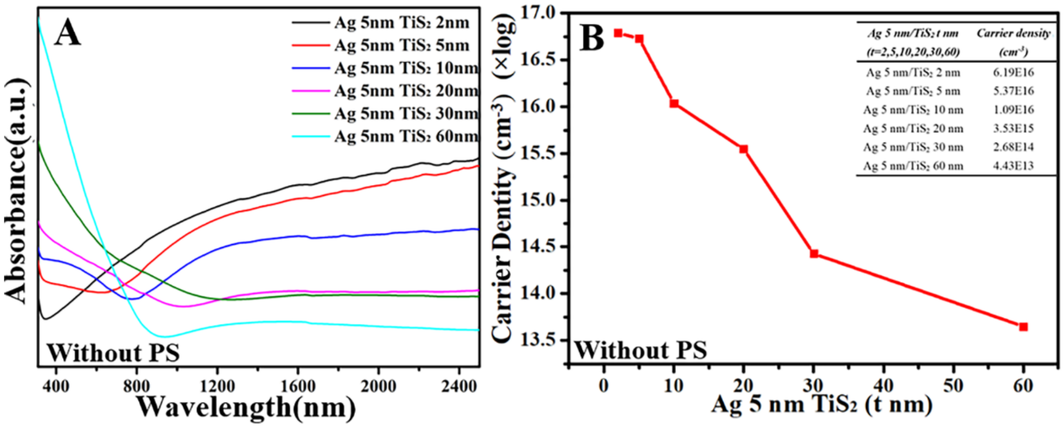

Figure S3. (A) UV-vis-NIR spectra and (B) Hall effect for Ag 5 nm/TiS_2 (t nm) nanostructures without PS, and the table of inset is the raw data.

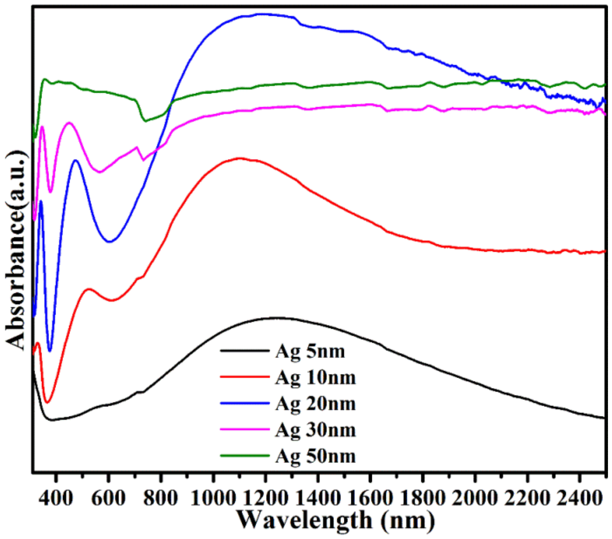

Figure S4. UV-vis-NIR spectra for pure Ag (t nm) layer nanostructures.
